# Application of Ganz Surgical Hip Dislocation Approach in Pediatric Hip Diseases

**DOI:** 10.4055/cios.2009.1.3.132

**Published:** 2009-08-17

**Authors:** Sung Jin Shin, Hong-Seok Kwak, Tae-Joon Cho, Moon Seok Park, Won Joon Yoo, Chin Youb Chung, In Ho Choi

**Affiliations:** Department of Orthopaedic Surgery, Seoul National University College of Medicine, Seoul, Korea.; * Department of Orthopaedic Surgery, Jeju National University College of Medicine, Jeju, Korea.

**Keywords:** Surgical hip dislocation, Femoroacetabular impingement, Pediatric hip disease

## Abstract

**Background:**

Ganz surgical hip dislocation is useful in the management of severe hip diseases, providing an unobstructed view of the femoral head and acetabulum. We present our early experience with this approach in pediatric hip diseases.

**Methods:**

Twenty-three hips of 21 patients with pediatric hip diseases treated using the Ganz surgical hip dislocation approach were the subjects of this study. The average age at the time of surgery was 15.7 years. There were 15 male and 6 female patients who were followed for an average of 15.1 months (range, 6 to 29 months). Diagnoses included hereditary multiple exostoses in 9 hips, slipped capital femoral epiphysis in 7, Legg-Calvé-Perthes disease in 4, osteoid osteoma in 1, pigmented villonodular synovitis in 1, and neonatal septic hip sequelae in 1. Medical records were reviewed to record diagnoses, principal surgical procedures, operative time, blood loss, postoperative rehabilitation, changes in the range of hip joint motion, and complications.

**Results:**

Femoral head-neck osteochondroplasty was performed in 17 patients, proximal femoral realignment osteotomy in 6, open reduction and subcapital osteotomy for slipped capital femoral epiphysis (SCFE) in 2, core decompression and bone grafting in 2, hip distraction arthroplasty in 2, and synovectomy in 2. Operative time averaged 168.6 minutes when only osteochondroplasty and/or synovectomy were performed. Hip flexion range improved from a preoperative mean of 84.7° to a mean of 115.0° at the latest follow-up visit. Early continuous passive motion and ambulation were stressed in rehabilitation. No avascular necrosis of the femoral head was noted up to the time of the latest follow-up visit, except for in one SCFE patient whose surgical intervention was delayed for medical reasons.

**Conclusions:**

Ganz surgical hip dislocation provides wide exposure of the femoral head and neck, which enables complete and precise evaluation of the femoral head and neck contour. Hence, the extensive impinging bump can be excised meticulously, and the circulation of the femoral head can be monitored during surgery. The Ganz procedure was useful in severe pediatric hip diseases and allowed for quick rehabilitation with fewer complications.

Most pediatric hip diseases are relatively benign compared with adult hip diseases, and are amenable to conservative treatment or extraarticular surgical procedures. However, in some clinical entities, a more aggressive approach is needed to preserve the hip joint as long as possible. Such entities include moderate to severe or unstable slipped capital femoral epiphysis (SCFE), Legg-Calvé-Perthes disease (LCPD) with severe residual deformity or of adolescent onset, and femoroacetabular impingement from extensive osteochondroma at the femoral neck. Diagnostic procedures with detailed physical examination, three-dimensional CT, and MR arthrography enable better diagnosis of acetabular and proximal femoral deformity or dysplasia in such patients. The open approach with surgical hip dislocation provides an unobstructed 360° view of the femoral head and acetabulum and also allows for better treatment.1) This approach has been applied for osteochondroplasty of femoroacetabular impingement (FAI),^2^,3) subcapital osteotomy for SCFE,4) and synovectomy. We report our early experience with the Ganz-type surgical hip dislocation approach for serious hip diseases in children and adolescents.

## METHODS

Twenty-three hips of 21 patients with pediatric hip diseases were treated with the surgical hip dislocation approach between December 2005 and February 2008. Fifteen patients were men and 6 were women. The mean age at the time of surgery was 15.7 years (range, 9.5 to 30.3 years). Three patients (4 hips) over 20 years of age in this series had an open physics at the time of diagnosis due to panhypopituitarism. Patients were followed for an average of 15.1 months (range, 6 to 29 months). Primary diagnoses included hereditary multiple exostoses (HME), SCFE, LCPD, osteoid osteoma, pigmented villonodular synovitis of the hip (PVNS) and neonatal septic hip sequelae.

The hip joint was approached as described by Ganz et al.1) Briefly, the patient was prepared and draped in the lateral decubitus position. A Kocher-Langenbeck or similar skin incision was made, and the fascia lata was split in line with it. A greater trochanter flip osteotomy was made, and the greater trochanter was retracted anteriorly along with the vastus lateralis and the gluteus medius. The interval between the gluteus minimus and the tendon of the piriformis was developed, and the gluteus minimus was retracted superiorly to expose the capsule. A z-shaped capsulotomy was made protecting the lateral retinacular arteries, and the hip joint was subluxated or dislocated anteriorly by flexion-external rotation-adduction. In cases with femoroacetabular impingement, all of which were cam-type impingement caused by focal or diffuse femoral head and neck deformity, osteochondroplasty was performed, contouring the head and neck junction following the normal femoral head contour and making the head-neck offset. Elimination of impingement was confirmed by dynamic assessment of the impingement. In patients over 12 years of age, osteochondroplasty was performed across the physis, if necessary, as the peripheral physeal arrest would cause only insignificant femoral head deformity in this age group, if at all. In one patient that was 9 years of age, the physis remained untouched as the impingement was caused by only the femoral neck mass.

We reviewed radiographs and medical records of the patients to determine diagnoses, principal surgical procedures, operative time, and postoperative rehabilitation. We also analyzed improvement in the range of hip motion in the sagittal plane. The complications of surgical dislocation were evaluated. Diverse surgical procedures for hip disease were combined with surgical hip dislocation including osteochondroplasty for impingement, proximal femoral osteotomy, synovectomy, and core decompression of the femoral head.

Twenty-three cases were classified into three categories according to surgical procedures peformed through surgical hip dislocation. Eight cases undergoing only osteochondroplasty and/or synovectomy comprised group A. Six cases undergoing additional procedures at sites other than the hip joint comprised group B. The remaining nine cases, which underwent additional hip procedures affecting hip range of motion or modifying postoperative rehabilitation, comprised group C, Operative time was analyzed in group A , while improvements in hip flexion range and the rehabilitation process were evaluated in groups A and B.

## RESULTS

Pertinent patient data are summarized in Table 1.

All nine HME hips showed femoroacetabular impingement due to sessile osteochondromas at the femoral neck. They were treated by osteochondroplasty via a surgical hip dislocation approach. In one patient, femoral varization-derotation osteotomy was combined, as coxa valga caused femoral head uncovering and acetabular dysplasia.

Five of seven SCFE patients were chronic with femoroacetabular impingement. Two patients with mild slips but evident impingement were treated using osteochondroplasty only. Three patients with moderate to severe slips underwent osteochondroplasty along with intertrochanteric (2 patients) or base-of-neck (1 patient) flexion-valgization osteotomy. The remaining two patients had unstable, severe slips and underwent open reduction of the femoral capital epiphysis along with subcapital cuneiform osteotomy through the surgical hip dislocation approach.

**Fig. 1:**
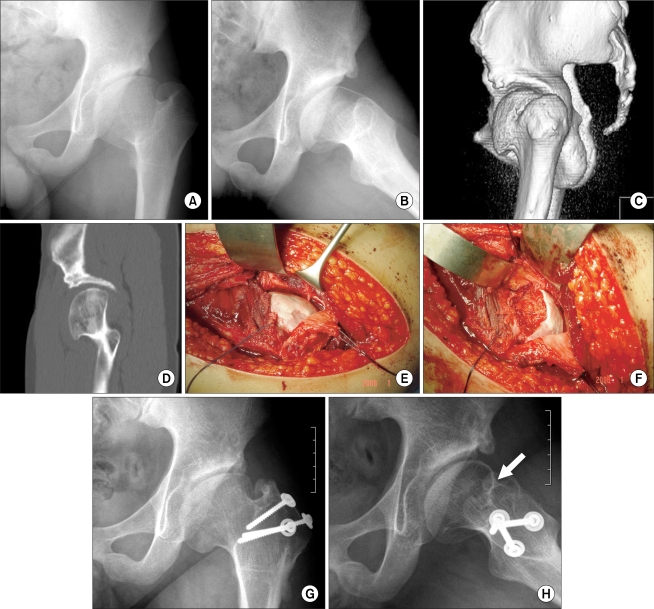
A 16-year-old boy with Legg-Calvé-Perthes disease of the left hip was treated with femoral valgization osteotomy 6 years ago and shelf acetabuloplasty 3 years ago. He had limited left hip flexion and a positive impingement test (A). Lauenstein view (B), 3D-CT (C), and sagittal reconstruction view (D) revealed an anterior hump of the femoral head causing cam-type femoroacetabular impingement, which was also confirmed in the subluxated femoral head using the Ganz surgical hip dislocation approach (E). Osteochondroplasty relieved the impingement (F). Plain radiographs taken 1.5 years later showed a well-formed head-neck offset (white arrow) (G, H). Hip flexion range improved from 95° in the preoperative period to 130° at 1.5 years follow-up.

Two of four LCPD patients showing FAI in the residual stage were treated by osteochondroplasty via a surgical hip dislocation approach with or without flexion-valgus osteotomy (Fig. 1). In the other two patients in early fragmentation stage, the inferomedial aspect of the femoral head was approached using surgical hip dislocation for core decompression, and hip distraction arthroplasty was applied.

One patient with an osteoid osteoma in the proximal femur had persistent hip joint synovitis and anterior femoral neck cortical thickening causing FAI. Through surgical hip dislocation, osteochondroplasty and synovectomy were performed.

One patient with PVNS was also treated by synovectomy through surgical hip dislocation. Another patient with neontatal septic hip sequelae (Choi's classification IIB) showed femoroacetabular impingement due to a bizarre anterior femoral head bump, which was excised using surgical hip dislcocation.

The operative time in group A (only osteochondroplasty and/or synovectomy group) averaged 169 minutes (range, 120 to 210 minutes). The range of hip flexion in groups A, and B improved from a preoperative value of 82.8° (range, 60 to 110°) to value of 108.9° (range, 90 to 130°) at the latest follow-up visit. Changes in hip flexion averaged 26.7° (range, 5 to 50°). Continuous passive motion (CPM) started an average of 2.2 days (range, 1 to 4 days) after surgery, and toe-touch ambulation started an average of 6.3 days (range, 3 to 10 days) after surgery in groups A and B. There was no increase in hip pain or stiffness after the surgical hip dislocation approach was adopted. No avascular necrosis of the femoral head was noted up to the time of the follow-up, except for in one unstable SCFE patient whose surgical intervention was delayed for a medical reason. There was no postoperative infection.

## DISCUSSION

Most pediatric hip diseases are treated by extra-articular procedures, such as soft tissue release, proximal femoral osteotomy, or pelvic osteotomy. However, sometimes a direct approach to the femoral head or acetabulum is indicated in serious conditions or residual stages in which further remodeling cannot be expected. The surgical hip dislocation approach proposed by Ganz et al.1) is very useful in such cases. This approach enables preservation of the femoral head blood supply and allows for a direct approach to the intra-articular lesion as well as the underlying pathology. Ganz et al.1) reported their experience using this approach in 213 hips over the course of seven years. Their cases represented femoroacetabular impingement resulting from abnormal femoral head-neck offset and non-spherical femoral head, PVNS, osteochondroma, and synovial chondromatosis. They believed that this technique could be combined with other procedures such as trochanteric advancement, relative femoral neck lengthening, and femoral neck osteotomy.

The hip joint can be surgically dislocated using other approaches. However, the Ganz method of surgical hip dislocation has several advantages. As the abductor is detached by trochanter flip osteotomy, rigid fixation of this flip fragment by screws restores immediate stability and allows for early mobilization of the patient. Moreover, by replacing this fragment to a point other than the osteotomy site, a trochanter transfer effect can be achieved if necessary. When flexion-valgization osteotomy is performed for SCFE patients, hip abductor alignment with the femoral shaft can be maintained by replacing the flip osteotomy fragment aligned with the distal fragment rather than the proximal one.

Hip arthroscopy is also gaining popularity in the management of hip diseases including femoroacetabular impingement.5) With accumulated experience, advancement in surgical skills and developments in instrumentation, arthroscopy might replace the surgical hip dislocation approach in the future. However, as surgical dislocation of the hip joint allows full access to the acetabular labrum, the acetabular cartilage and the whole proximal aspect of the femur,2) it is currently well-indicated for extensive femoroacetabular impingement or complex hip disorders as in this series. Surgical hip dislocation also enables more accurate contouring of the femoral head-neck junction and dynamic observation of the impingement. Indications, advantages and disadvantages of surgical hip dislocation in comparison with hip arthroscopy need to be further studied.

The surgical hip dislocation approach is a major surgical procedure, taking more than 2 hours even when only osteochondroplasty is performed. Blood loss was enormous in some cases of this series because excision of the sessile type osteochondroma left a wide, bleeding cancellous bone surface. We advocate the use of a cell-saver in such a situation. However, meticulous hemostasis could limit the amount of bleeding per se.

Although this approach involves extensive dissection around the hip joint, and even the femoral head is partially or completely dislocated, postoperative rehabilitation is quite fast for the dimension of surgery. Our protocol is to mobilize the hip joint using a CPM machine on postoperative day 3 and to encourage partial weight-bearing on day 7 as long as the bony integrity of the femur was not compromised by femoral osteotomy. All the patients without proximal femoral osteotomy or distraction arthroplasty in this series could follow this protocol. Krueger et al.6) reported that persistent pain in patients with surgical dislocation of the hip but no evidence of cartilaginous or osseous alterations, this pain could have resulted from intra-articular adhesions. We did not observe such phenomena in any patients in this series. It is highly likely that early CPM prevented intra-articular adhesions and promoted early recovery.

Orthopaedic surgeons who are not familiar with this approach may be concerned about the possibility of avascular necrosis of the femoral head. However, accurate knowledge of vascular anatomy around the hip joint relieves the surgeon. The deep branch of the medial femoral circumflex artery (MFCA) runs along the greater trochanter anterior to the short rotators, and is safely protected as long as the trochanter flip osteotomy is placed posterior to the short rotators.1) The lateral retinacular braches enter into the lateral aspect of the femoral head, which can be protected by a precise z-shaped capsulotomy. Anterior dislocation of the femoral head does not influence the natural course or tension of the extracapsular deep branch or intracapsular branches of the MFCA, as long as the obturator externus is left attached.7) Wide exposure of the femoral head and neck and subsequent dissection of the lateral retinaculum under direct vision and palpation make this procedure safer than other approaches, which provide only limited exposure of the susceptible anatomical structures. Moreover, the femoral head blood circulation can be monitored during the procedure by making a 2 mm drill hole. This step is not always necessary, but serves as a very useful tool in marginal procedures such as subcapital osteotomy and open reduction of unstable slipped capital femoral epiphysis. Among the 23 cases of surgical hip dislocation approach in our series, only one case showed postoperative avascular necrosis of the femoral head. Surgical intervention in this case was delayed for a week for medical reasons, and the femoral head was found to be avascular intraoperatively. Therefore, it is highly likely that the delayed intervention caused avascular necrosis rather than the surgical hip dislocation approach itself.

## Figures and Tables

**Table 1:**
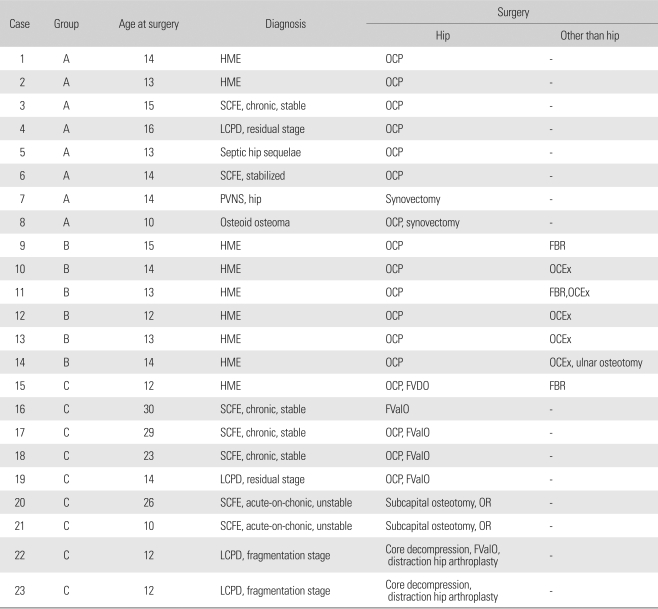
Pertinent Data of the 23 Cases

Cases 16 and 17 are both hips of the same patient. HME: Hereditary multiple exostoses, OCP: Osteochondroplasty, SCFE: Slippled capital femoral epiphysis, LCPD: Legg-Calvé-Perthes disease, FVDO: Femoral varization-derotation osteotomy, FValO: Femoral flexion-valgization osteotomy, OR: Open reduction, FBR: Metallic foreign body removal, OCEx: Osteochondroma excision.
